# Ecological Momentary Assessment and Alcohol Use Disorder Treatment

**DOI:** 10.35946/arcr.v36.1.10

**Published:** 2014

**Authors:** Jon Morgenstern, Alexis Kuerbis, Frederick Muench

**Affiliations:** Jon Morgenstern, Ph.D., is a professor of psychology and Alexis Kuerbis, L.C.S.W., Ph.D., L.M.S.W., is an assistant professor of social work, both in the Department of Psychiatry, Columbia University Medical Center, New York. Frederick Muench, Ph.D., is a clinical psychologist and the director of digital health interventions in the Department of Psychiatry at North Shore Long Island Jewish Health System in New York and an adjunct at New York University’s Interactive Telecommunications Program, New York.

**Keywords:** Alcohol use, abuse, and dependence, alcohol use disorders (AUDs), assessment, assessment methods, ecological momentary assessment (EMA), real-time assessment, feedback, mobile technologies, mHealth, literature review

## Abstract

The ability to capture real-time data on human behavior inexpensively, efficiently, and accurately holds promise to transform and broaden our understanding of many areas of health science. One approach to acquiring this type of real-time data is ecological momentary assessment (EMA). This method has been used to collect data in many domains of addiction research, including research on the treatment of alcohol use disorders (AUDs). Empirical evidence supports the hypothesis that use of EMA can improve the quality of AUD treatment research when compared with standard assessment methods because it provides more accurate reporting, allows investigators to examine the dynamic unfolding of the behavior change process at an individual level, and can be used to augment and improve clinical assessment and treatment. Overall, the existing literature provides strong support for the advantages of EMA when combined with standard assessment of addictive behaviors in general. Nevertheless, use of EMA in AUD treatment research thus far has been limited, especially in the area of research on mechanisms of behavior change. Existing research indicates, however, that EMA can be used to deliver tailored feedback as a novel and potentially transformative approach to improving AUD treatment. This research area clearly warrants additional future efforts.

Ecological momentary assessment (EMA) involves repeated sampling of individuals’ behaviors and experiences in real-time, in the individuals’ natural environment (see article by Arora in this issue). Whereas early EMA studies used paper diaries, recent developments in mobile technologies now enable EMA-based studies to use smartphones equipped with increasingly sophisticated sensors that can passively measure such variables as geolocation, physical activity, and heart rate. The ability to capture real-time data on human behavior inexpensively, efficiently, and accurately is poised to transform and broaden our understanding of many areas of health science. As a result, there has been a dramatic increase in the use of EMA as a research tool over the last decade (Mehl and Conner 2012; Stone et al. 2007). The primary aim of this article is to examine EMA in the context of alcohol treatment research. Specific topics addressed include what types of research questions or treatments have been studied using EMA, whether these studies have yielded new knowledge regarding critical treatment constructs or improved treatment outcomes, and what lessons can be drawn from EMA research that can inform future studies.

The article addresses these questions by focusing on three areas where EMA is thought to confer an advantage over standard assessment methods, including (1) more accurate or unbiased reporting of behavior and experience; (2) the ability to examine the dynamic unfolding of behavior change processes within individuals; and (3) the ability to extend observation or intervention from the clinic to the natural environment, thereby augmenting clinical assessment or treatment. For each of these areas, the article briefly will describe the potential advantage of EMA, present studies that illustrate how the issue has been evaluated, and summarize findings to date with a focus on clarifying how EMA has advanced our understanding of AUD treatment. This review is not designed to provide an exhaustive overview of all available studies but seeks to illustrate the types of studies that have been conducted and the knowledge gained. Although the focus here is on treatment for alcohol use disorders (AUDs), EMA research on other addictive behaviors, notably nicotine addiction, has on occasion advanced further than it has in the AUD arena. Thus, when appropriate, the article will describe EMA studies of other addictive behaviors and discuss how they might be applied to AUD treatment. Finally, the article will summarize the current status of EMA research in AUD treatment and offer several recommendations for future work.

## EMA and Reporting Accuracy

EMA is thought to substantially improve accuracy of reporting compared with global, lab-based self-report measures. With certain research questions (e.g., in studies of relapse), standard self-report sometimes requires participants to recall events over lengthy periods. Such recall may introduce a systematic bias that distorts accurate reporting. In addition, standard assessments often ask individuals to aggregate or summarize their experiences. Aggregation of subjective states (e.g., cravings) or cognitive processes (e.g., self-efficacy), especially when in a laboratory setting, is likely to introduce some level of error. The accuracy of EMA has been compared with standard self-report measures using three types of approaches:

- EMA analyses of drinking have been compared to calendar methods, such as timeline follow-back (TLFB) interviews (Sobell et al. 1996) to assess drinking outcomes.
- EMA of cognitive, affective, or motivational processes have been compared with standard measures of similar constructs.
- Retrospective recall of relapse has been compared with real-time EMA of these events.

All three types of studies have found discrepancies between standard measurement and EMA, but the degree of divergence varied depending of the phenomena being examined.

### EMA vs. Calendar Methods in Drinking Outcomes

A handful of alcohol treatment studies (Kranzler et al. 2004, 2014; Lincoln et al. 2011) have compared real-time and calendar methods to assess drinking outcomes. Kranzler and colleagues (2004) assessed nine participants seeking AUD treatment using TLFB and daily interactive voice recording (IVR) during a 12-week treatment trial. Results indicated poor correspondence between the two approaches on measures comparing the amounts participants drank on specific days, even when comparing a 2-week TLFB recall period to IVR. An aggregate measure of drinking showed better correspondence, but IVR yielded a significantly higher level of drinking on average than did the 12-week TLFB recall. Similarly, Searles and colleagues (1995 Similarly, Searles and colleagues (2002) found that respondents significantly underestimated their alcohol consumption using timeline methods compared with daily IVR in 1-and 3-month outcomes. This discrepancy was significantly more pronounced among people with higher alcohol consumption (Searles et al. 2000). In a similar study, Lincoln and colleagues (2011) compared IVR and a 6-week TLFB of drinking outcomes for 28 participants in AUD treatment. The results showed poor agreement in recall of daily drinking patterns; however, unlike the studies by Kranzler and colleagues (2004) and Searles and colleagues (1995 and Searles and colleagues (2002), the research of Lincoln and colleagues (2011) yielded no significant differences between the two approaches with respect to aggregate drinking measures. Finally, Kranzler and colleagues (2014) conducted a set of outcome analyses using both TLFB and IVR drinking outcomes and found no differences in clinical trial results. These findings generally are consistent with the larger literature comparing real-time and calendar methods in community and college-student samples (Shiffman 2009).

Taken together, these findings suggest that although calendar methods appear to be less accurate in capturing day-to-day variations in drinking patterns and may underestimate consumption, especially in cases of longer recall periods, they seem to be adequate for capturing aggregate measures of drinking outcome. In addition, it is important to note that reliance on IVR alone to assess drinking outcomes puts investigators at risk of missing data if there is any inconsistency in IVR compliance; TFLB data, in contrast, are relatively complete. Thus, even AUD treatment studies that use IVR to assess outcome tend to augment their analyses with TLFB (Morgenstern et al. 2012).

Combining multiple data collection methods such as baseline laboratory measurements and EMA has several advantages. It may improve our understanding of how trait measurements interact with dynamic process variables collected through EMA, leading to better understanding of certain mechanisms of change (Shiffman et al. 2008). It can also help create more reliable methods of data collection for different populations. For example, in an analysis by Patrick and Lee (2010), three different methods of data collection resulted in different measurements of consumption that were further influenced by moderator variables, such as gender. The combination of data collection methods using multiple mediums also will become more commonplace as mobile and wireless alcohol sensors become more reliable and less invasive (Leffingwell et al. 2013). Methods such as transdermal alcohol sensors and mobile phone–based blood alcohol concentration (BAC) calculators, breath-based alcohol measurements, speech analysis, and infrared spectroscopy (Marques and McKnight 2007) are being developed and tested. Such methods hold promise to significantly improve investigators’ ability to accurately assess alcohol consumption, understand the determinants of risky drinking, and trigger real-time interventions. As these methods of data collection become more reliable, the ability to capture real-time information-process determinants will help build more accurate models of change. Although an in-depth discussion of these methods is beyond the scope of this article, it is important to note that these newer methods also require significantly greater data-management and analysis expertise than do self-report methods. Similarly, factors such as technology outages, user burden, and poor understanding of proper assessment schedules (e.g., fixed vs. variable) represent new challenges to the integration of mobile methods into alcohol research.

### Testing Putative Process Theories

Until recently, virtually all empirical tests of putative links between process determinants, mediators or moderators, and alcohol treatment outcomes have been examined using standard aggregate measures. For example, the hypothesized link between self-efficacy and outcome has generally been assessed using standard self-report measures that ask participants to recall their self-efficacy during a period of several weeks and then aggregate these ratings to arrive at a composite index. A handful of alcohol studies have compared EMA and questionnaire methods to assess putative process variables, but only one of those was conducted in an AUD treatment-seeking population. The study compared standard measures of self-efficacy and readiness to change with daily IVR measures of these constructs in a sample of 89 participants seeking AUD treatment (Kuerbis et al. 2013). The investigators aggregated the daily scores of the IVR variables into a single index for the week prior to randomization and compared that index with the standard pretreatment measures of readiness and self-efficacy to assess their agreement and ability to predict drinking outcomes during an 8-week treatment period. The results indicated only modest agreement across methods. Moreover, IVR measures of readiness and self-efficacy significantly predicted drinking outcomes, whereas standard measures did not.

Several studies have used EMA methods to probe the hypothesized relationship between drinking-to-cope (DTC) motives and real-time relationships between negative mood and drinking in community samples. DTC theories (Cooper et al. 1992) posit that relief of stress and negative affect is a powerful determinant of drinking and that the potency of this motive differs across individuals. Studies have used EMA methods generating real-time reports of drinking and affects to examine whether people scoring high on a DTC questionnaire show stronger relationships between stress or negative affect and drinking (Armeli et al. 2008, 2010; Piasecki et al. 2014; Todd et al. 2005). These studies have yielded substantially weaker support for the DTC hypothesis than prior cross-sectional studies. Generally, the results suggested that although DTC questionnaires tap some individual differences in drinking motives, the relationship between dispositional motives, proximal mood or stress, and drinking is much more complex than anticipated, suggesting the need for substantial revision of drinking-motive theory (Shiffman 2009).

EMA approaches also can be used to investigate relapse processes. Relapse theories have had a pivotal influence on the treatment of addictive disorders, including AUDs (Marlatt and Gordon 1986; Witkiewitz and Marlatt 2007). Until the mid-1990s, research on relapse was based on retrospective recall of relapse events, many of which took place weeks or even months prior to data collection. Shiffman and colleagues (1996, 1997) conducted several seminal studies examining the influence of recall bias on the reports of putative relapse processes in smoking. These studies compared retrospective recall of smoking lapse and relapse with real-time monitoring of similar processes using electronic diaries among smokers seeking to quit smoking. Results indicated that agreement between recall and real-time report of lapses was quite poor. In addition, contrary to existing relapse-theory hypotheses, neither negative affective reactions to lapse and feelings of guilt nor decreases in self-efficacy predicted progression from a lapse to a relapse. Surprisingly, no similar studies of relapse have yet been conducted for AUD treatment.

Overall, evidence supports the advantages of EMA in terms of reporting accuracy over standard laboratory assessment methods, which have been the mainstay of AUD clinical research. The limitations of standard assessment methods are especially notable in assessing cognitive, affective, or motivational processes. When taken together with studies conducted on other addictive behaviors (Shiffman 2014), the studies that have assessed EMA approaches in AUDs suggest that the real-time assessment of process variables can counterbalance a number of the existing limitations to global report methods and lead to substantial revisions in theories of predictors, mediators, and moderators of AUD treatment (Riley et al. 2011).

## EMA and Temporal Unfolding of Within-Individual Change Processes

Because EMA allows for collection of frequent, repeated measures of individuals’ thoughts and behaviors over time, it provides a powerful tool for examining within-person change processes. In addition, EMA is able to capture contextual events and, thus, can facilitate the exploration of person-by-context interactions. As a result, EMA enables researchers to describe and analyze the unfolding of sequences of experiences and events as they play out over time. Shiffman and colleagues (2009) have described this feature of EMA research as analogous to a “movie” that shows dynamic relationships as they unfold, whereas global or recall methods can be likened to still photography that provides a static single-shot representation of what is essentially a dynamic phenomenon.

In a series of seminal studies, Shiffman and colleagues (2005) used EMA to test the dynamic role of negative affect and self-efficacy in smoking relapse. The study design included two novel features enabled by EMA. First, relapse was represented as a sequence of conditional events that began with a triggering event or high-risk situation, which in turn led to either a highly tempting situation (experience of craving but no smoking) or a lapse. The lapse then led to either a relapse or a return to abstinence. Second, factors influencing relapse were ordered based on their dynamic properties. Thus, they were classified as either stable (e.g., gender), tonic or slow moving (e.g., stress build-up), or momentary (e.g., rapid change in negative affect). Contrary to relapse theory, tonic relapse factors, such as higher levels of stress or negative affect in the days immediately prior to the lapse/relapse episode did not significantly predict a lapse. By contrast, momentary factors, such as rapid increases in negative affect in the minutes or hours before the episode did predict a lapse. In addition, the link between negative affect and a lapse seemed to be moderated by a person-level factor: nicotine dependence severity. Thus, people with more severe dependence were more likely to lapse in the context of negative affect, whereas people with less severe dependence were more likely to lapse in the context of drinking alcohol. Analyses of self-efficacy and lapse revealed a similar set of complex interrelations among person-level factors, slow-moving background factors, momentary influences, and contextual events as predictors of a return to smoking.

Only a handful of studies have examined dynamic features of relapse as predictors in AUD treatment using EMA (Chih et al. 2014; Collins et al. 1998; Cooney et al. 2007; Holt et al. 2011). These studies all examined the momentary influence of predictors on lapse by assessing these factors in the period immediately prior to the lapse event, while controlling for baseline levels of the same factors. For example, Holt and colleagues (2012) examined dynamic changes in affective states, urge, and self-efficacy in the hours before a first lapse to drinking among participants in concurrent alcohol and smoking cessation treatment. Constructs were assessed at baseline and then repeatedly during treatment using random and event-based prompts to assess dynamic change in a prospective design. Contrary to study hypotheses, only the urge to smoke among those who had smoked already significantly predicted lapse to drinking. Although results differed across the studies, none of the analyses supported negative affect and urge as momentary predictors of lapse in alcohol treatment. A few other studies have used daily IVR to examine the role of affective states, urge, and self-efficacy in alcohol treatment (Armeli et al. 2006; Kranzler et al. 2004). However, these studies are limited in their ability to fully assess the temporal relationships between precipitants of consumption and drinking, in part because they measured same-day rather than lagged relationships.

Overall, a large and comparatively sophisticated literature on smoking cessation (see Shiffman 2014) illustrates the novel ability of EMA both to capture and analyze the temporal unfolding of hypothesized sequences of experiences and events within individuals and to probe complex person level-by-context interactions. In addition, studies have begun to examine the relationship between momentary influences and relapse in illicit drug users in treatment (Epstein and Preston 2010; Epstein et al. 2009). In contrast, EMA approaches and their features to date have received little attention in the AUD treatment literature. The lack of EMA studies in AUD treatment relative to smoking cessation likely reflects early concerns among researchers that AUD clinical populations may not be able to manage relatively expensive electronic diary devices and provide reports when intoxicated. Recent feasibility studies among illicit drug users indicate, however, that these problems are surmountable, especially given the growing use of smartphones (Epstein et al. 2009).

Another important factor in the slow uptake of EMA methods to study change process in AUD treatment research may be a failure to fully appreciate the value of well-conducted EMA studies in improving AUD treatment. Programmatic research by Shiffman and colleagues (2005, 2008) on the dynamic interaction of processes in smoking cessation has revealed two central findings, both of which have far reaching implications for addiction treatment research. These findings relate to substantive advances in understanding relapse as a dynamic and complex phenomenon with individuals struggling to regain and maintain self-control over addictive behaviors and to the match between theory and method in behavior change research (Riley et al. 2011; Sterba and Bauer 2010; Tan et al. 2012).

### Relapse As a Dynamic and Complex Phenomenon

As mentioned previously, EMA research on smoking cessation has identified the heightened importance of proximal or momentary influences in the relapse process (Shiffman 2005). For example, affective processes may be highly variable, exhibiting changes in the span of minutes or even seconds. Such sudden changes in mood or rapid depletion of self-control resources have been shown to predict relapse (Brandon et al. 2007). Similarly, rapidly changing contextual factors (e.g., being offered a cigarette by a friend) also play an important role in relapse. Although prior conceptualizations identified cognitions, affects, and situations as relapse predictors, these factors were largely seen as slow moving or tonic. Current conceptualizations, in contrast, view relapse as a process occurring over time, where stable traits and slow-moving background factors (e.g., stress) create a vulnerability to relapse. These factors then interact with momentary influences to trigger relapse (McKay et al. 2006; Shiffman et al. 2009).

This revised perspective suggests the importance of research on momentary influences on the behavior change process as a strategy to improve AUD treatment. By definition, momentary influences can be difficult to predict. In addition, they often occur outside of the individual’s awareness. EMA studies—including those that assess factors such as implicit cognitions—are needed to fully understand the unfolding of behavior change processes (Marhe et al. 2013) and identify critical junctures as temporal targets for interventions. Smartphones include numerous features that can aid in the assessment of explicit and implicit influences on behavior. The assessment of objective parameters, such as context and location sensing, physiology, speech, sleep, and activity among others, have tremendous potential to help researchers understand the mechanisms of behavior change (Bacon 2013; Dulin et al. 2013, 2014; Gustafson et al. 2014; Scharnweber et al. 2013; Vahabzadeh et al. 2010). Other methods used in general health behavior change, such as qualitative journaling and ecological video journaling (Melton and Bigham 2013) also provide real-time methods to improve understanding of clients in their everyday lives.

Research on momentary influences and relapse suggest that helping people monitor implicit and explicit processes in real time and using this information to deliver interventions at critical moments in the natural environment might improve AUD treatment outcomes (Ebner-Premier and Trull 2009; Shiffman et al. 2008). Accordingly, EMA-enabled research on the dynamics of change processes in AUD treatment will help improve our understanding of the mechanisms of behavior change and thus allow us to improve treatment.

### Treatment Theory–Method Match

Recent discussions of behavior change research methods have demonstrated the importance of using methods that adequately capture the dynamic and complex nature of most behavior change processes (Collins 2006; Sterba and Bauer 2010; Tan et al. 2012). AUD treatment theories posit that interrelationships among stable patient characteristics, internal states, and environmental contexts predict drinking and that these interrelationships change as a result of treatment and time. Moreover, the temporal dynamics of critical constructs likely vary substantially. For example, the impact of stressful events on drinking likely is cumulative and occurs over days or weeks and may account for fewer than expected findings on the relationship between momentary stress and drinking. By contrast, the impact of craving on drinking likely occurs within seconds or minutes. From a methods perspective, real-time, intensive longitudinal assessment that matches the temporal resolution of the hypothesized relationships is necessary to adequately test AUD behavior change theories. Appropriately selected EMA methods allow for the collection of information with sufficient detail to provide discriminating tests of AUD treatment theories.

Shiffman and colleagues (2008) have referred to research that examines the interplay of motivational, cognitive, affective, and behavioral processes as they unfold over time as the study of “microprocesses.” These investigators note that insight into microprocesses potentially will have a major impact on improving behavioral interventions because such insight helps identify leverage points in treatment. In fact, EMA’s ability to enable this type of research may be its most important contribution to clinical psychology. Nevertheless, several relatively challenging methodological issues associated with using EMA remain as researchers strive to understand intra-individual change and translate this knowledge into timely and context-sensitive interventions.

## Using EMA to Augment AUD Clinical Assessment and Treatment

EMA tools are increasingly being incorporated into behavioral intervention, an approach that has been called ecological momentary intervention (EMI) (Heron and Smyth 2010). EMIs are characterized by the delivery of interventions to people during the course of their everyday lives (i.e., real time) and in their normal settings (i.e., real world). EMIs can take many forms, from a patient receiving a text message as part of an alcohol intervention (Muench et al. 2014; Suffoletto et al. 2012, 2014) to the delivery of long-term care management for AUDs using a smartphone application that is linked to clinical support (Gustafson et al. 2014). The development of EMIs or mHealth interventions is a rapidly evolving area, and a comprehensive review is beyond the scope of this article (for more information, see the article by Beckjord and Shiffman in this issue). Instead, this section will focus on the role of real-time or ambulatory assessment in the delivery of EMIs and, more specifically, their utility in tailoring treatments.

EMA and EMIs have several features that could improve AUD treatment. Given problems associated with recall bias, real-time assessment could improve the accuracy of clinical assessment and treatment planning. EMA also could be used to reduce burden and increase compliance with self-monitoring of symptoms—an important component of most behavioral interventions—even over lengthy periods. In addition, self-monitoring across behavior-change interventions is associated with improved outcomes (Heron and Smyth 2011), including improved alcohol use outcomes. For example, Dulin and colleagues (2014) found that participants rated the alcohol-tracking feature in a smartphone application for problem drinking as the most helpful feature. Moreover, these authors found that more intensive use of the smartphone application was associated with improved outcomes, results that correspond to Web-based alcohol research literature (Cunningham et al. 2011).

In addition to self-monitoring, many AUD treatments involve some skills training with the expectation that patients will practice and master those skills in their natural environments. EMI could be used to provide such in vivo skills training (Dulin et al. 2014; Gustafson et al. 2014). EMI could further be used to personalize or tailor treatment in two ways. First, information collected during real-time assessments could be used to provide tailored feedback to patients either at a single point in time or repeatedly over the course of treatment (Riley et al. 2011). Second, feedback could be individually timed to match a predetermined context, such as a high-risk situation or subjective state (e.g., craving) (Gustafson et al. 2014). Given the dynamic and momentary nature of relapse precipitants, the ability to intervene in the moment would add an important component to AUD treatment that could dramatically improve outcomes.

The ability to tailor interventions in a just-in-time setting can be seen as a natural extension of adaptive treatments— that is, treatments that are successively modified based on response to a prior stage of the intervention (McKay et al. 2009). This type of EMI has been called a just-in-timeadaptive intervention (JITAI). The widespread use and multiple technological features of today’s smartphones provide a resource-rich platform for delivering JITAIs. As noted above, smartphones are equipped with passive data collection capabilities that can substantially diminish the burden of data collection, provide virtually continuous monitoring and increase the amount and type of information available to generate feedback. Although a number of technological obstacles remain, a critical scientific challenge in developing JITAIs is how to translate the wealth of real-time information available into effective personalized, timely, and context-sensitive feedback.

### Examples of EMA-Augmented AUD Treatments

Litt and colleagues (2009) used EMA to assess high-risk situations and coping response in a study of the effectiveness of coping skills training. The investigators hypothesized that one reason for the apparent lack of evidence for a specific therapeutic effect of a commonly used treatment approach—cognitive behavioral therapy (CBT)—may have been the failure of manual-driven CBT to accurately assess and intervene with a patient’s specific coping-skills deficit. Participants were asked to use cellphones in the 2 weeks prior to treatment to record their urges, coping responses, and drinking behavior as they occurred. This idiographic information on drinking antecedents was summarized and then provided to therapists who used the feedback to tailor their skills training. Participants were randomly assigned to the individualized assessment and treatment program (IATP)–CBT condition or to standard, manualized CBT (SCBT). IATP–CBT yielded a higher proportion of abstinent days, more momentary coping, and less drinking in high-risk situations than SCBT. These findings provided one of the earliest examples of how EMA can be used to tailor treatments and improve their efficacy.

In a recent study, Gustafson and colleagues (2014) reported on the efficacy of a continuing-care EMI intervention for AUD patients transitioning from residential care. The EMI was a multi-feature smartphone application based on self-determination theory called Addiction-Comprehensive Health Enhancement Support System (A-CHESS). It was designed to provide continuous real-time monitoring and support during early recovery and included internet data access to deliver static educational content as well as interactive features, such as a GPS-activated alert that automatically warned patients when they entered a high-risk situation. In addition, patients completed a Web-based weekly survey (Weekly Check-In) on A-CHESS that assessed drinking over the prior week, as well as a set of items designed to assess relapse risk (e.g., relationship problems) and protective factors (e.g., AA meeting attendance). A randomized clinical trial comparing A-CHESS to standard continuing care found that A-CHESS yielded significantly lower rates of drinking over a 12-month period (Gustafson et al. 2014). (For more information on the A-CHESS application and its evaluation, see the article by Quanbeck et al. in this issue.)

These two examples demonstrate how EMA has been used to tailor AUD interventions. In IAPT, EMA data was collected prior to treatment, summarized, and provided to the clinician who then used this information to develop a personalized treatment plan. In A-CHESS, EMA data was collected repeatedly over the extended treatment period, and a predictive model iteratively determined the probability of weekly relapse risk based on a cumulative record of patient lapse history and current functioning. The A-CHESS feedback could be adjusted weekly based on current risk categorization and delivered to the patient in his natural setting. Several other technology-based EMI systems currently are being developed, such as the Location-Based Monitoring and Intervention System for Alcohol Use Disorders (LBMI-A) (Dulin et al. 2014) and the Scandinavian combined Web-and mobile-based alcohol intervention (Brendryen et al. 2014). Researchers also are testing an adaptive text-messaging intervention for problem drinking that adapts weekly to the user’s self-reported goal achievement using EMA (Muench et al. 2014), highlighting that even simple technologies available on every phone can be used to develop adaptive interventions.

### Challenges to the Development of Personalized,Timely, and Context-Sensitive AUD Interventions

One obstacle to the future development of JITAIs is that the behavior-change theories that underlie AUD treatment have provided limited guidance in prior efforts to tailor treatments (Morgenstern and McKay 2007). The development of any JITAI requires an understanding of how stable patient characteristics interact with momentary subjective states and contextual factors to predict intra-individual change. As noted above, studies on the temporal unfolding of behavior change processes indicate that current theories are either inaccurate or inadequately specified to provide a framework for such predictions (Riley et al. 2011; Shiffman et al. 2005).

A related challenge is the use of standard statistical approaches to analyzing the temporal unfolding of multiple factors within individuals, which can be assessed using intensive longitudinal data. Standard methods have significant limitations in testing theories about complex and time-varying interactions that occur within individuals. For example, standard methods such as multilevel modeling aggregate individuals under the assumption that groups share a similar set of change processes (Sterba and Bauer 2010). However, this assumption may be erroneous because examining interactions at a group level (i.e., determining average change) may have little to do with what happens for an individual (Bolger et al. 2013; Molenaar 2004). Similarly, standard methods are limited in their ability to model nonlinear and time-varying interactions among variables (Tan et al. 2012; Walls and Shafer 2006). Overall, researchers are recognizing that new efforts to revise behavior-change theory, coupled with the novel analytic approaches, will be needed to inform the development of JITAI (Mohr et al. 2013; Riley et al. 2011; Tan et al. 2012; Timms et al. 2014). (See also the article by Beckjord and Shiffman in this issue.)

One novel and promising direction towards meeting these goals is to conceptualize behavior-change processes as a complex, dynamic system (Resnicow and Vaughn 2008; Witkiewitz and Marlatt 2007) and to use analytic approaches such as mathematical modeling and control engineering to develop JITAIs for behavioral problems, including AUDs (Banks et al. 2014; Riley et al. 2011; Rivera 2007). This approach has been used successfully to develop adaptive interventions in people with HIV (Rosenberg et al. 2007). With this approach, complex dynamic systems are characterized as possessing multiple factors that interact dynamically and change over time. The components of such systems are highly interconnected, such that each influences the others, often in nonlinear ways. Moreover, relationships between elements of the system can be short-lived and characterized by positive-and negative-feedback loops. Finally, the system’s functioning is influenced both by its cumulative history (i.e., prior characteristics) and by current context (Marewski and Olsson 2009).

Several empirical studies (Hufford et. 2003; Witkiewitz et al. 2007) have supported the hypothesis that relapse is a highly complex process characterized by nonlinear dynamics. A recent study by Banks and colleagues (2014) used mathematical modeling of dynamic systems to examine behavior change processes among 89 problem drinkers in AUD treatment, using daily EMA. These analyses provided strong support for the conceptualization of behavior change as a dynamic nonlinear process and illustrated the limitations of using standard approaches to examine intra-individual change using EMA data. Although the results were promising, however, the investigators noted that research in this area still is in its early stages.

## Summary and Future Directions

EMA is widely considered to represent a major advance in assessment methodology because of its ability to increase the accuracy of reporting, enable the examination of the dynamic unfolding of behavior change processes within individuals, and augment clinical assessment and treatment (Mehl and Connor 2013). The studies reviewed in this article support these advantages for addictive behaviors in general. Given these advantages, it is surprising that EMA has not been used more widely in AUD treatment research. Only a handful of studies have compared the accuracy of global self-report with that of EMA for drinking outcomes. These studies suggest that global measures like the TLFB yield similar findings to EMA for aggregate measures of drinking outcome, but are less effective at capturing day-to-day variation in drinking patterns.

Reporting bias seems to be even more problematic for global measures assessing cognitive, motivational, affective processes than for measures of behavior (Shiffman 2009). The few AUD treatment studies reviewed above suggest similar limitations for constructs representing global measures of change processes, such as drinking motives, motivation to change, and self-efficacy. The overwhelming majority of AUD treatment studies to date have used global measures rather than EMA to assess change processes. These studies have addressed critical aspects of AUD treatment, including hypothesized treatment moderators and mediators. Findings reviewed above suggest that the true limitations of standard methods to assess change processes may be underappreciated. More research is needed that allows for comparison of EMA and global self-report measures to determine whether better measurement of change processes might lead to substantive modifications in understanding the change process, especially regarding moderators and mediators of AUD treatment. In addition, the combination of multiple methods and media of data collection has significant advantages over single methods, and more research should be conducted with a variety of assessment types when feasible.

The use of EMA to study the temporal unfolding of behavior change represents a major methodological advance in efforts to understand mechanisms underlying behavior change. EMA allows investigators to capture events and experiences with a high degree of temporal resolution and to probe the interrelationship of multiple factors within an individual over time. As noted above, current treatment theories implicitly postulate that behavior change represents the cumulative influence of multiple, time-varying influences that occur within an individual. However, standard research methods have limited our ability to represent and test dynamic, complex interactions. Surprisingly few studies have used temporal unfolding designs to examined AUD treatment, and even these studies were limited to testing the relationship between a single dynamic factor and relapse.

The development of EMI or mHealth interventions represents a promising and rapidly evolving area. EMI offers new features compared with standard interventions, including the ability to deliver tailored feedback based on ambulatory assessment. Several AUD treatment studies have incorporated novel EMA approaches to deliver tailored feedback, and the results demonstrate the potential for this approach to improve AUD treatment. The technological sophistication of smartphones with multimodal assessment capabilities suggests that this may be a feasible platform for a new and previously difficult-to-imagine form of personalized treatment through the provision of automated tailored feedback. Development of JITAIs for AUDs will require a substantially stronger empirical knowledge base regarding the mechanism of behavior change. The research on the temporal unfolding of behavior change in smoking cessation represents an important step in that direction, but further novel advances in theory building and methods are needed to adequately capture the complex and dynamic nature of behavior change processes and translate this process into actionable feedback.
